# ELISA based on a recombinant *Paragonimus heterotremus* protein for serodiagnosis of human paragonimiasis in Thailand

**DOI:** 10.1186/s13071-018-2878-5

**Published:** 2018-05-30

**Authors:** Kanokkarn Pothong, Chalit Komalamisra, Thareerat Kalambaheti, Dorn Watthanakulpanich, Timothy P. Yoshino, Paron Dekumyoy

**Affiliations:** 1 Department of Helminthology, Faculty of Tropical Medicine, Mahidol University, Bangkok, 10400 Thailand; 2 Mahidol-Bangkok School of Tropical Medicine, Faculty of Tropical Medicine, Mahidol University, Bangkok, 10400 Thailand; 3 Department of Microbiology and Immunology, Faculty of Tropical Medicine, Mahidol University, Bangkok, 10400 Thailand; 4 Department of Pathobiological Sciences, School of Veterinary Medicine, University of Wisconsin, Madison, Wisconsin 53706 USA

**Keywords:** *Paragonimus heterotremus*, cDNA library, Recombinant protein, Paragonimiasis, Immunodiagnosis, IgG-ELISA

## Abstract

**Background:**

*Paragonimus heterotremus* is the main causative agent of paragonimiasis in Thailand. In Western blot diagnostic assays for paragonimiasis, the 35 kDa band present in crude *P. heterotremus* somatic extracts represents one of the known diagnostic bands. This study aimed to use a *P. heterotremus* cDNA library to create a recombinant version of this antigen for use in immunodiagnosis of paragonimiasis.

**Methods:**

To accomplish this aim a cDNA expression library was constructed from adult worm mRNA and immuno-screened using antibodies from mice that had been immunized with the 35 kDa antigen. Screening resulted in the identification of an immunoreactive protein encoded by clone CE3, which contained an inserted sequence composed of 1292 base pairs. This clone was selected for use in the construction of a recombinant *P. heterotremus* protein because of its similarity to proactivator polypeptide. For recombinant protein expression, the CE3 gene sequence was inserted into the plasmid vector pRset and the resulting product had the expected molecular weight of 35 kDa. An IgG-ELISA based on the CE3 recombinant protein was evaluated by using sera from healthy individuals, from patients with paragonimiasis and other parasitic infections. This ELISA was performed by using human sera diluted at 1:2000, an optimized antigen concentration of 1 μg/ml, and anti-human IgG diluted at 1:4000.

**Results:**

The cut-off optical density value was set as the mean + 2 standard deviations (0.54), which resulted in the test having a sensitivity of 88.89% and a specificity of 95.51%. The recombinant antigen could react with antibodies from *P. heterotremus*, *P. pseudoheterotremus* and *P. westermani* infections. Cross-reactivity occurred with a few cases of *Blastocystis hominis* infection (2/3), Bancroftian filariasis (1/10), opisthorchiasis (3/10), strongyloidiasis (4/10) and neurocysticercosis (1/11).

**Conclusions:**

Given the high test sensitivity and specificity, reflected in the low level of heterologous infection cross-reactivity (11/215 serum samples), observed in the IgG-ELISA, this 35 kDa antigen may be useful for the detection of paragonimiasis.

**Electronic supplementary material:**

The online version of this article (10.1186/s13071-018-2878-5) contains supplementary material, which is available to authorized users.

## Background

*Paragonimus* species, also known as lung flukes, are the causative agents of pulmonary paragonimiasis in humans. In Asia, the occurrence of paragonimiasis is 90%, with about 20 million individuals infected [1, 2]. Seven species of the genus *Paragonimus* have been recorded in Thailand: *P. bangkokensis*, *P. harinasutai*, *P. heterotremus*, *P. pseudoheterotremus*, *P. siamensis*, *P. westermani*, and *P. macrorchis*. Of these, *P. heterotremus*, *P. pseudoheterotremus* and probably *P. westermani* are reported in human cases [3–5]. People become infected with *Paragonimus* parasites when they frequently eat improperly cooked freshwater crabs containing *Paragonimus* metacercariae collected from mountainous streams.

Several suspected cases of paragonimiasis have been clinically identified without the detection of *Paragonimus* eggs or worms in the sputum, feces, or tissues. Notably, the ectopic foci of this worm in various host tissues can result in cerebral, cutaneous and other clinical forms of paragonimiasis that can be difficult to diagnosis. Therefore, immunodiagnosis is an important supplement to the parasitological methods used in the detection of *Paragonimus* infections [6, 7]. Specifically, immunoblotting tests using *P. heterotremus* crude extracts of adult worms show high sensitivity and specificity to paragonimiasis heterotremus with diagnostic bands corresponding to 32.5, 33, and 35 kDa native antigens [8] and can differentiate between *P. heterotremus* and *P. westermani* Korean strain [9]. Another *P. heterotremus* component, a 31.5 kDa partially purified antigen, also has been used in the diagnosis of human paragonimiasis [10]. Molecular techniques have been applied as a tool for producing recombinant protein antigens to replace the native antigens produced by other parasitic worm species from animal hosts in similar tests [11]. Obtaining *Paragonimus* adult worms for native antigen preparation is a very time-consuming and costly process which involves crab collection, recovery of metacercariae from crabs, infecting and maintaining mammalian definitive hosts, confirming infections by eggs detection, and finally obtaining worms from host lungs and processing them for diagnostic testing. Therefore, the aim of the present study was to apply a molecular approach to generate *P. heterotremus* DNA recombinant antigens for use in immunodiagnosis of human paragonimiasis. To this end, we constructed a complementary DNA (cDNA) library from adult *P. heterotremus* worms and used a molecular cloning approach to identify and express recombinant proteins that exhibited selective immunoreactivity with antibodies recognizing the 32.5, 33 and 35 kDa bands comprising *P. heterotremus* native antigens. Herein we report the DNA cloning, identification and expression of a recombinant protein for the 35 kDa antigen and the construction of an IgG-ELISA (enzyme-linked immunosorbent assay) based on this protein. Results of testing against a panel of human sera indicate that the recombinant protein generated by this study may be useful in the serodiagnosis of human paragonimiasis.

## Methods

### Worm collection

The metacercariae were recovered from freshwater crabs (*Larnaudia larnaudii*), which were collected from paragonimiasis endemic areas of Nakon Nayok Province, central Thailand. Gerbils were infected by feeding five fresh metacercariae to each animal using a stomach tube connected to a syringe and maintained for at least 35 days, at which time *P. heterotremus* eggs were observed in the feces using a simple smear technique. Gerbils were then euthanized and the adult worms isolated from the lungs and surrounding lung tissues. Isolated worms were washed 3 times in 0.85% sterile NaCl and used for preparing crude antigen and for isolation of total worm RNA.

### Construction and immunoscreening of the cDNA library

To construct the cDNA, total RNA was extracted from fresh *P. heterotremus* adult worms by using the single-step method of total RNA extraction with TRIzol® reagent (Invitrogen-Life Technologies, Carlsbad, CA, USA). Messenger RNA (mRNA) was isolated using a solid-phase oligo-dT matrix (Oligotex mRNA mini kit; Qiagen, Hilden, Germany) according to the manufacturer’s instructions. Two micrograms of mRNA template was used in the Universal RiboClone® cDNA Synthesis System (Promega, Madison, WI, USA) and further ligated into a pJET 1.2/blunt Cloning Vector (CloneJET PCR Cloning Kit, Thermo Fisher Scientific, Rockford, IL, USA). The ligation mixture was used directly to transform *Escherichia coli* XL1-Blue (Stratagene, Agilent Technologies, Santa Clara, CA, USA) competent cells, followed by plating on LB agar plates supplemented with ampicillin for Ampicillin-selection. To identify the antigen-producing clones by immunoscreening, the pooled negative (pre-immunized sera) and positive sera from mice that had been immunized with the crude eluted 32.5, 33 and 35 kDa antigens of *P. heterotremus* were used to identify clones carrying the 32.5, 33 and 35 kDa antigens. Positive colonies were identified by aligning the membrane with the agar plates. For DNA sequencing, the PCR was performed by using pJET 1.2 forward and pJET 1.2 reverse sequencing primers. The resulting gene sequences were searched against the NCBI database.

### Target sequence amplification

The protein targets for DNA cloning were selected based on the results of immunoscreening tests, aimed to extend the target sequences. The L_2F was selected as target clone. The designed primers were: Ph3 forward primer 5'-CTC TAG AAG ATC TCC TAC-3' and Ph3 reverse primer 5'-AGC CGA ACG ACC GAG CGC-3'. After PCR amplification, the PCR product was further purified. The purified DNA was cloned, designated clone CE3, by using a StrataClone PCR Cloning kit (Stratagene, Agilent Technologies), according to the manufacturer’s instructions.

### Construction of a recombinant protein clone

#### Plasmid creation

Primers for clone CE3 were designed based on the DNA sequencing results. These include: CE3 forward primer, 5'-CGA ACG TCG AGC GCT TAC TGT GAC A-3', which contained *Xho*I restriction enzyme sites, and CE3 reverse primer, 5'-AGC CGA GAA TTC GAG CGC CTT GCA AAA G-3', which contained *Eco*RI restriction enzyme sites. To amplify the target protein, PCR was performed as described above. Both the pRsetB vector (Invitrogen Ltd., Paisly, UK) and the purified DNA were digested with *Xho*I and *Eco*RI restriction enzymes using the Fermentas FastDigest Restriction Enzymes kit (Thermo Fisher Scientific). Finally, ligation was performed, and the obtained plasmid was transformed into *E. coli* host cells (strain DH5α or strain DE3 BL21 pLysS).

#### Protein expression and purification of the recombinant protein

A single colony of CE3 protein-expressing host cells was inoculated in a tube that contained LB broth with ampicillin and chloramphenicol and further incubated overnight at 37 °C with shaking at 180 rpm (New Brunswick Scientific Innova® 40, Edison, NJ, USA). The cultured cells were then inoculated into ampicillin-chloramphenicol LB broth and shaken at 37 °C for 3 h. IPTG (1 mM isopropyl-l-thio-β-D-galactopyranoside) was added to induce protein expression and shaken at 180 rpm for an additional 4 h at 37 °C. The cultured cells were centrifuged for the pellet and stored at -70 °C until required.

Following recombinant protein induction, host cells were pelleted and then extracted by suspending cells in BugBuster™ (Novagen™, Merck, Darmstadt, Germany). The extract mixture was centrifuged for collecting the supernatant containing solubilized proteins. The putative recombinant His6 (Histidine 6) fusion protein was then purified using a Ni-NTA (nickel-nitrilotriacetic acid) chromatography column according to the manufacturer’s instructions (Ni-NTA ProBond, Invitrogen, USA). Eluted fractions were collected and checked by SDS-PAGE analysis for the desired protein. Eluted fractions that possessed a single protein band were pooled, subjected to dialysis, and lyophilization.

#### Immunoreactivity of purified recombinant proteins to sera of immunized mice

Three mice were each injected *via* the intraperitoneal route (IP) with 1 μg of the 35 kDa recombinant protein, mixed 1:1 with Inject® Alum (Thermo Fisher Scientific). Mice were given IP booster injections with the same dose twice more at 2-week intervals. Blood samples were collected at 1 week after the last injection, and the resulting sera were tested against each recombinant protein antigen by Western blot analyses.

### Evaluation of recombinant proteins for serodiagnosis of paragonimiasis heterotremus

#### Human sera

The collection of patients’ sera used as the diagnostic test panel in the present study was stored specimens that had been used in the previous serological surveys. All samples had been maintained at -80 °C in the Department of Helminthology, Faculty of Tropical Medicine, Mahidol University for several years, and contained no patient identifying information (see Ethical Approval and Consent section). Details of the diseases and corresponding diagnostic methods used on the groups of serum samples (Group A: paragonimiases, Group B: other parasitic infections and Group C: healthy controls) for the evaluation of the recombinant antigens are shown in Table 1.

**Table 1:** Diseases, and corresponding diagnostic methods, detected in the serum samples

Group	Diseases	No. of serum samples	Diagnostic methods
A	Paragonimiasis heterotremus	29	Worm and egg detection, serum samples collected for over 25 years, patients resided in 2 endemic provinces of *P. heterotremus* in the central Thailand where metacercariae from crabs were identified by PCR a few years ago
	Paragonimiasis pseudoheterotremus	3	Eggs in feces and PCR (1 case), immunoblot detection and PCR for detection of metacercariae from crabs (2 cases). Patients resided along Thai-Myanmar border
	Paragonimiasis westermani	4	Serum samples were supported by Korean researcher
	Total	36	
B	Gnathostomiasis	10	Worm and immunoblot detection
	Strongyloidiasis	10	Larva detection
	Hookworm infection	10	Egg detection
	Trichinellosis	10	Larva and immunoblot detection
	Capillariasis	3	Egg, larva, adult worm detection
	Toxocariasis	10	Immunoblot
	Angiostrongyliasis	10	Worm detection, immunoblot
	Ascariasis	6	Egg and worm detection
	Trichuriasis	9	Egg and worm detection
	Trichostrongyliasis	10	Egg detection
	Bancroftian filariasis	10	Microfilariae detection
	Enterobiasis	4	Egg and worm detection
	Brugian filariasis	10	Microfilariae detection
	Brugian filariasis	10	ELISA using recombinant antigen
	Dirofilariasis	2	Worm detection
	Neurocysticercosis	11	Cyst detection, immunoblot
	Sparganosis	6	Sparganum detection
	Taeniasis	13	Egg or segments (*T. solium* or *T. saginata*)
	Echinococcosis	3	Protoscolices detection
	Hymenolepiasis nana	5	Egg detection
	Hymenolepiasis diminuta	1	Egg detection
	Opisthorchiasis	10	Worm detection
	Echinostomiasis malayanum	1	Egg detection and PCR
	Fascioliasis	3	Egg detection, immunoblot
	Minute intestinal flukes infections	10	Worm detection
	Creeping eruption	3	Symptoms, negative for strongyloidiasis and gnathostomiasis
	Entamebiasis	4	Cyst detection
	Giardiasis	3	Cyst detection
	*Blastocyctis hominis* infection	3	Cyst detection
	Falciparum malaria	5	Blood stage detection
	Vivax malaria	5	Blood stage detection
	Tuberculosis	5	Acid fast stained *Mycobacterium tuberculosis*
	Total	215	
C	Healthy serum	30	Negative ELISA using ten kinds of antigens and fecal examinations
	Total	30	

*Key*: Group A, paragonimiases; group B, heterologous infections; group C, healthy controls

#### Enzyme-linked immunosorbent assay (ELISA)

An indirect ELISA was created based on the recombinant 35 kDa protein antigen and was evaluated using groups of sera from patients infected with paragonimiases (*n* = 36), heterologous infections (*n* = 215) and healthy controls (*n* = 30). The ELISAs were performed as described by Dekumyoy et al. [8] with minor modifications. Single serum dilutions, 1:2000, of individual positive and negative sera against 1 μg/ml purified recombinant antigen were performed to establish the optimal conditions for the tests. In addition, 1:4000-diluted horseradish peroxidase-conjugated rabbit anti-human IgG (Southern Biotech, Birmingham, USA) and colormetric substrate solution [ABTS, 2, 2-azino-di-(3-ethyl-benzthiazoline sulfonate); Sigma, Oakville, Ontario, Canada] were used. The reactions were stopped by adding 50 μl of 1% SDS solution. Antigen-antibody binding reactions were measured by reading the OD_405_ (optical density) using an ELISA Reader (Tecan, Männedorf, Switzerland).

### Data analysis

The ELISA cut-off point was chosen by using receiver operating characteristic (ROC) curve analysis, sensitivity *versus* 1-specificity or false positive. The SPSS version 18 was used as tool for ROC curve analysis. All data were further analyzed by using the Mann-Whitney U-test. The calculation was carried out on SPSS version 18 and a *P*-value < 0.05 was considered as significant. The accuracies of the diagnostic test, including sensitivity, specificity, and predictive values, were determined using the method of Parikh et al. [12] and SPSS version 18 and MedCal (free statistical calculators, diagnostic test evaluation calculator) were used at 95% CI. The ELISAs were analyzed by calculating the means and standard deviations (SD). A cut-off value at 0.543 was done by ROC curve analysis and the mean + 2SD. The AUC was 0.975.

## Results

### Isolation of total RNA, purification of mRNA, and construction of a *P. heterotremus* cDNA library

A double-stranded *P. heterotremus* cDNA reaction was run on a 1.2% agarose gel along with a 1 kb DNA size marker. The sizes of the cDNA bands ranged from 0.5 to 12 kb. After the cDNA was purified by using Promega Wizard® DNA Clean-UP System (Promega, Madison, WI, USA), the double-stranded cDNA was successfully ligated into the pJET 1.2/blunt cloning vector and used to transform in *E. coli* XLI Blue on LB agar. In order to check the size of inserts, 14 colonies were randomly picked and PCR was performed. The sizes of insert cDNAs, as determined by performing colony PCR, ranged from approximately 500 to 1000 bp (Additional file 1: Figure S1). The products of the positive clones that were larger than 500 bp were further assessed by colonies amplification and sequencing.

### Sequence analysis

We determined the DNA sequences of each of the 11 plasmid clones described above, the insert sizes of which ranged from 500 to 1000 bp. Homology searches against NCBI databases (https://www.ncbi.nlm.nih.gov/) revealed some clones matched were a trematode group i.e. clone IDs, P89-1, P51-R, P55-R, P3_1F. The identities of these matches are listed in Table 2, and the percentage of identity of these eleven clones ranged from 31 to 100% when searched against the trematodes, bacteria and protozoans in the NCBI database.

**Table 2:** The sequence analysis results after being searched against NCBI databases

Sample No.	Clone ID	Name	Percentage of identity
1	P89-1	Fatty acid-binding protein type 3 (*Clonorchis sinensis*)	70
2	P51-F	Microcystin synthetase (uncultured *Microcystis* sp.)	73
3	P51-R	Dynein beta Chain ciliary (*C. sinensis*)	97
4	P55-R	Hypothetical proteinSMP_137720 (*Schistosoma mansoni*)	31
5	P3_1F	SJCH6C65074 protein (*Schistosoma japonicum*)	78
6	P7_2UF	Histone lysine methyltransferases (*Toxoplasma gondii*)	43
7	L_2F	Hypothetical protein (*Echinococcus multilocularis*)	92
8	L_3R	Axonemal beta dynein heavy chain, putative (*T. gondii ME49*)	37
9	L_2F2	Hypothetical protein (*Plasmodium chabaudi chabaudi*)	99
10	L_8F	Protein Bm11556, partial (*Brugia malayi*)	100
11	L3R	Hypothetical protein Emj 000005500 (*E. multilocularis*)	92

### PCR confirmatory test and DNA cloning

After the initial cloning described above, the inserted cDNA that was > 500 bp in size was run on a 1.2% agarose gel. The band containing the inserted cDNA was then cut and purified for further second cloning to extend sequences. Eleven plasmid clones were identified by immuno-screening of the cDNA library. The inserted cDNA from clone L_2F (designated CE3) was run on a 1.2% agarose gel, and based on comparison with the Kapa Universal ladder, was approximately 900 bp long (Additional file 2: Figure S2). The CE3 clone was selected as representative of the antigenic clones since it possessed the longest sequence that contained no stop codons.

### Sequence analysis

**Fig. 1:**
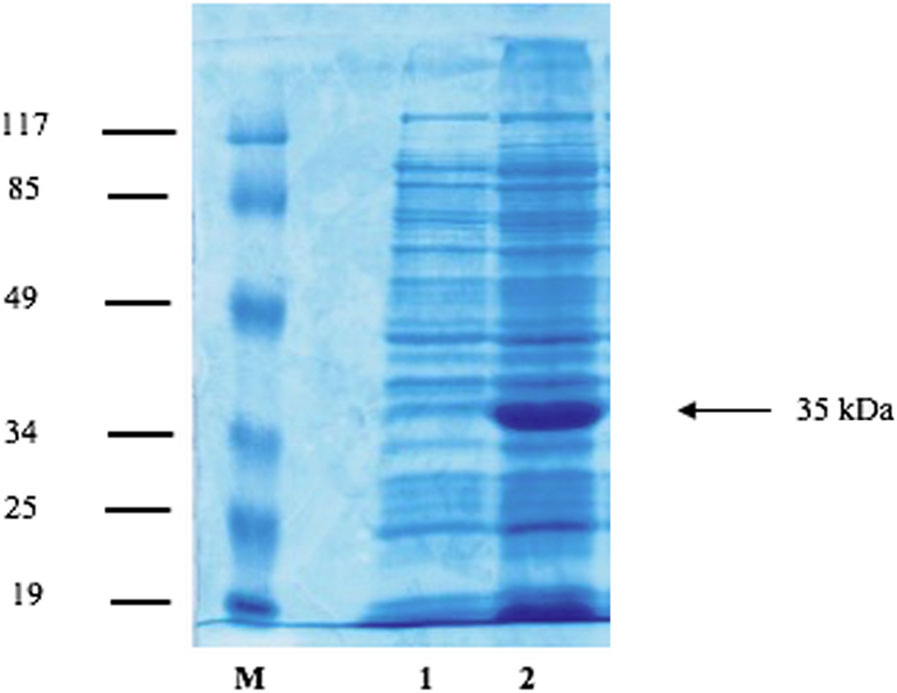
Assessment of recombinant fusion protein production in CE3-clone transformed *E. coli* cells. The proteins from these cells were separated on a 12% gel by SDS-PAGE and stained with Coomassie blue. Lane M: molecular weight markers; Lane 1: non-IPTG-induced CE3 cells; Lane 2: IPTG-induced CE3 cells. Arrow indicates the prominent band at 35 kDa

After the DNA sequences were checked for open reading frames and stop codons, clone number 3 (CE3) was chosen for recombinant protein production since CE3 was given the similar identity of sequences to other clones and had a long sequence without stop codon between the sequences. Specific primers were used to amplify the DNA *via* PCR. A sequence analysis of the recombinant protein from clone CE3 and the corresponding alignment scores calculated by the NCBI-Blastx program show that it shares identity with the proactivator polypeptides of *C. sinensis* and *Schistosoma haematobium* at 48 and 29%, respectively (Table 3). An overview of the CE3 clone using DNAMan software consists of 1292 bp with an open reading frame at 49–1047 bp (Additional file 3: Figure S3), under the accession number KX180136, and the estimated molecular weight of the corresponding protein is 35 kDa (Fig. 1).

**Table 3:** Alignment score of the putative amino acid sequences of the 35 kDa antigen as calculated by the NCBI-BLASX program from clone CE3

Accession No.	Sequence description	Identity (%)	Score (Bits)	*E*-value
GAA56617.1	Proactivator polypeptide (*Clonorchis sinensis*)	48	297	4E-92
XP_012799469.1	Proactivator polypeptide (*Schistosoma haematobium*)	29	197	3E-34
AAW27625.1	SJCHGC01869 protein (*Schistosoma japonicum*)	28	237	1E-31
CCD59012.1	Saposin-containing protein (*Schistosoma mansoni*)	27	245	1E-31

### IPTG-induced protein expression and purification of recombinant proteins

We examined the effect of IPTG-induction on recombinant protein expression. The IPTG-induction was started from 0 h (non-IPTG-induced) to 3 h (IPTG-induced); proteins were observed every hour. The protein expression was increased following timing. Whole bacterial lysates from non-IPTG-induced and IPTG-induced CE3 clones at 3 h were analyzed by SDS–PAGE in a 12% polyacrylamide gel and stained with Coomassie brilliant blue. After IPTG induction for 3 h, there was an increase in the amount of an expressed 35 kDa protein (Fig. 1), indicating that the fusion protein from clone CE3 was successfully expressed in *E. coli* upon IPTG induction.

After extraction of bacterial cells with BugBuster™ and centrifugation and washes with lysis buffer (8M urea in LEW buffer), the crude recombinant protein extract was subjected to passage through a nickel-agarose affinity column, followed by elution with buffer containing various concentrations of imidazole ranging from 100 to 500 mM. A SDS-PAGE analysis of the purified protein fractions eluted at imidazole concentrations of 25, 50, 100, 200 and 500 mM showed the expected enrichment of the 35 kDa band in the eluted fractions. The isolated fractions recovered from the 100, 200 and 500 mM imidazole elutions were then pooled and dialyzed against 0.1× PBS (phosphate buffered saline-Tween20 buffer, pH 7.2) for further use.

### Mouse immunization with the 35 kDa recombinant protein

**Fig. 2:**
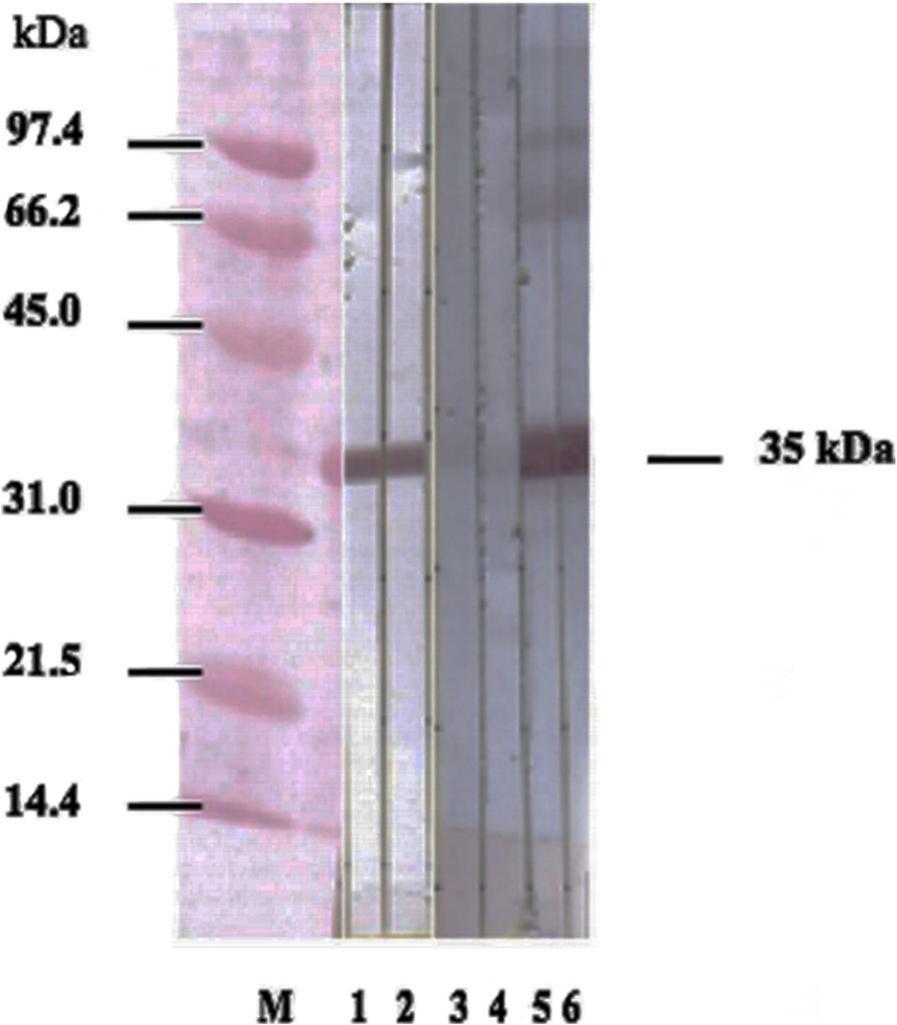
Reactivity of mouse anti-CE3 clone against the CE3 recombinant protein. The reactivity of serum samples from mice immunized with the recombinant protein from CE3 clone against CE3 recombinant protein was assessed by Western blot analysis. His-tagged CE3 recombinant protein was blotted with anti-his tag antibodies (Lanes 1, 2), normal (non-immunized) mouse sera (Lanes 3, 4), or anti-CE3 clone antibody (Lanes 5, 6). Lane M: protein molecular weight standards

After isolation, the 35 kDa purified recombinant protein was used to immunize mice for specific antibody production. ELISAs were performed against the purified recombinant protein. The mouse antibody against the 35 kDa recombinant protein had titer over 1:1600. Additionally, using Western blot analysis, the 35 kDa recombinant protein from the CE3 clone also was recognized by the antibodies in the sera of immunized mice, but not by sera from normal, unimmunized mice (Fig. 2).

### Serodiagnostic evaluation of the 35 kDa recombinant protein by ELISA

**Fig. 3:**
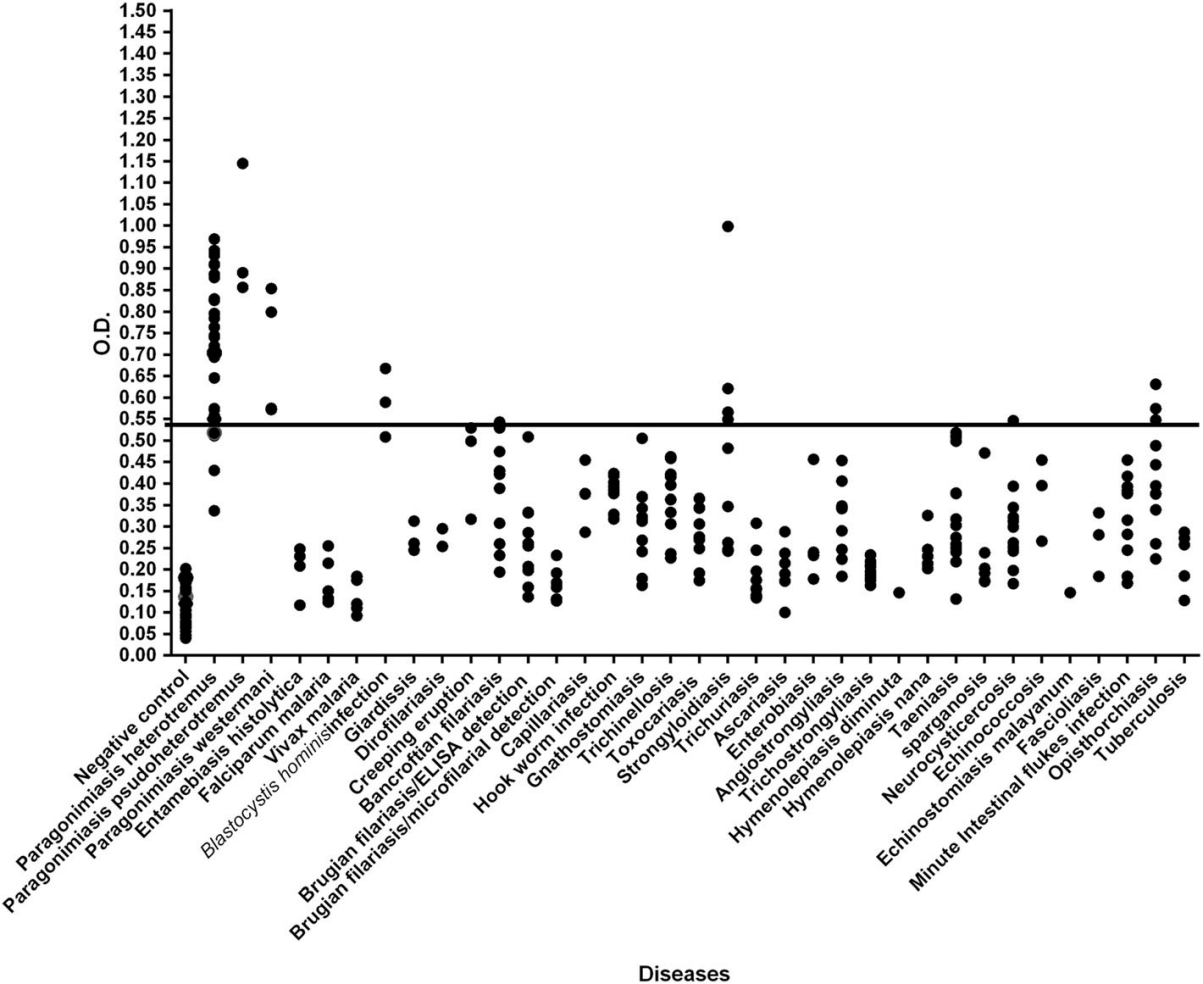
Scatter pattern of ELISA absorbance values was developed by using purified recombinant antigen, and the specificity of this assay was assessed using serum samples from paragonimiasis patients, healthy human controls (negative control) and patients with various parasitic and bacterial diseases (cut-off at a mean + 2 SD = 0.54)

An indirect ELISA was developed using the 35 kDa recombinant protein and sera from healthy controls or from patients with human paragonimiasis or other parasitic infections (Table 1) were used to assess the specificity and sensitivity of this assay as an immunodiagnostic tool. The ELISA conditions were optimized by fixing the concentration of the secondary antibody at a 1:4000 dilution and then optimizing the primary antibody and recombinant protein concentrations. As a result, in subsequent assays, the primary antibodies (human sera) were diluted to 1:2000, and 1 μg/ml of recombinant protein was used to coat wells of ELISA plates. Based on the ELISA results from 30 negative serum samples, the cut-off value was selected from the mean ± 2 SD at OD_405_ = 0.54. Evaluating the IgG-ELISA with this cut-off value produced sensitivity, specificity, and positive and negative predictive values at 88.89, 95.51, 74.42 and 98.32%, respectively **(**Table 4**)** (calculated by MedCal with 95% CI). Based on this OD_405_ cut-off value of 0.54, 32 of 36 serum samples from patients with confirmed paragonimiasis gave positive assay results. The 4 false negative sera, although below the 0.54 OD threshold, were not far from the cut-off value. Regarding to paragonimiasis, this recombinant antigen strongly react with serum antibodies from 3 cases of paragonimiasis pseudoheterotremus and 2 cases of paragonimiasis westermani in the high ODs-ranges, 0.856–1.145 and 0.799–0.853, respectively. The antigen weakly reacted with antibodies from tuberculosis. Importantly, this recombinant antigenic protein resulted in true negative readings for sera representing 27 of 32 different parasitic diseases, as well as for all negative controls. Cross-reactivity occurred for five diseases (11/215 serum samples), including *Blastocystis hominis* infection (2/3), Bancroftian filariasis (1/10), opisthorchiasis (3/10), strongyloidiasis (4/10), and neurocysticercosis (1/11). We observed that one of four false positives for strongyloidiasis gave a quite high OD-value, above the cut-off value. The OD-values of false positives from *Blastocystis hominis* infection, Bancroftian filariasis, neurocysticercosis and opisthorchiasis were close to the cut-off value **(**Fig. 3).

**Table 4:** Cut-off values, sensitivity, specificity, and positive and negative predictive values of the *P. heterotremus* 35 kDa recombinant fusion protein ELISA. The ELISA was performed using 1 μg of *P*. *heterotremus* 35 kDa recombinant fusion protein and 30 negative serum samples diluted at 1:2000. Test results were determined by the OD_405_ values of all serum samples under the cut-off value of 0.54

Cut-off value	OD at 405 nm	Sensitivity (%)	Specificity (%)	Predictive values (%)	
				Positive	Negative
Mean **+** SD	0.44	80	80	80	80
Mean **+** 2 SD	0.54	100	100	100	100
Mean **+** 3 SD	0.64	0	100	0	50
Evaluation of test under the cut-off value in this study					
Mean **+** 2 SD	0.54	88.89	95.51	74.42	98.32

### Statistical analysis

All data were analyzed by SPSS version 18 and were found to be non-parametric. A value of P < 0.0001 was considered as significant (Wilcoxon signed-rank test: *Z* = 30365.000, Mann-Whitney U-test: *U* = 230, *Z* = -9.306, *P* < 0.0001). The statistical results indicated that there was a significant difference between paragonimiasis samples and non-paragnimiasis samples, heterologous infected groups and healthy control. Moreover, the median scores of the paragonimiasis and non-paragonimiasis groups were 257.78 and 123.94, respectively.

## Discussion

In the present study, a novel recombinant protein antigen was produced from a cloned cDNA of *P. heterotremus* worms, designated CE3. This insert cDNA sequence exhibited closest homology to a proactivator polypeptide of the liver fluke, *Clonorchis*, and the encoded a protein antigen that specifically reacted to antibodies directed against the specific 35 kDa diagnostic band seen in native adult worm antigen extracts. The IgG-ELISA used to evaluate this recombinant protein yielded sensitivity, specificity, and positive and negative predictive values of 88.89, 95.51, 74.42 and 98.32%, respectively, and resulted in false positives for only four helminthic infections and one protozoan infection. Specificity of the test using this recombinant antigen is quite high, which was evaluated against 32 different diseases as indicated above. It should be noted by including serum samples from patients infected with the three human *Paragonimus* spp: *P. heterotremus*, *P. pseudoheterotremus* and *P. westermani*, we found that the ELISA was not able to discriminate between species-specific infections. However, given the similarities in infection transmission, clinical disease and treatment options for these lung fluke infections, having a broad-based diagnostic for human paragonimiasis, regardless of the species, provides a valuable tool for infection screening and/or diagnoses. The expressed recombinant *P. heterotremus* protein (CE3), when incorporated into the ELISA immunoassay, not only exhibited the expected high specificity to antibodies from mice immunized with the recombinant 35 kDa protein, but also showed positive reactive and high specificity to antibodies from 32 of 36 human paragonimiasis serum samples. As noted, the other four paragonimiasis sera produced the OD-values close to the cut-off point. Antibody cross-reactivity is largely related to the presence of helminth protein epitopes that are shared in common with similar proteins from other parasite species resulting in a false positive test outcome, or the individual donor may have been previously infected with lung flukes. However, only a few cases of other parasitic diseases, opisthorchiasis, strongyloidiasis, Bancroftian filariasis, neurocysticercosis and *B. hominis* infection cross-reacted with the proactivator polypeptide recombinant antigen. Considering location of these parasites in patients, as is generally known, *B. hominis* is an intestinal protozoan and mostly asymptomatic, *Opisthorchis viverrini* is located in the bile duct and *Wuchereria bancrofti* is located in the lymphatic. Symptoms typically seen in infections by all three of these parasites are quite different from lung fluke infections, and therefore, can be more easily diagnosed based on clinical presentations. However, *S. stercoralis* larvae pass the lungs during their development before moving to the intestine to become adult worms, but most OD-values were lower than the cut-off value. The serum sample exhibiting cross-reactivity above the cut-off may be a result from a co-infection with *Paragonimus* because it has been previously shown [8] that antibodies produced in naturally-acquired opisthorchiasis, strongyloidiasis, and neurocysticercosis do not react with 35 kDa antigen of adult *P. heterotremus* worm. We noted in the study of Yoonuan et al. [13] that antibody cross-reactivity of opisthorchiasis, ascariasis and strongyloidiasis also occurs in IgG-ELISA using 35 kDa cathepsin L lacking signal peptide (rPpsCatL, recombinant *P. pseudoheterotremus* cathepsin L). However, although both the proactivator polypeptide (current study) and the cathepsin L proteins share a similar 35-kDa molecular mass, both appear antigenically distinct based on differences in their immunoreactivity profiles. Also our antigen did not cross-react with ascariasis patient sera, further distinguishing it from the recombinant cathepsin L.

It is known that serodiagnosis can play an important role as a supplementary method of human paragonimiasis diagnosis. An ELISA is a complementary method for diagnosis of cerebral paragonimiasis, chronic cerebral paragonimiasis, pulmonary and ectopic pulmonary paragonimiasis [14, 15]. In 1991, Indrawati et al. [6] produced a partially purified adult worm antigen of *P. heterotremus* using Sephadex column chromatography and subsequently used this antigen preparation in the serodiagnosis of human paragonimiasis heterotremus. The IgG–ELISA constructed using this antigen showed 100% sensitivity and 100% specificity (seven diseases). A partially purified antigen has been prepared from adult worms of *P. heterotremus* using an isoelectric focusing cell (Rotofor, Bio-Rad, Hercules, California, USA). This partially purified antigen shows a specific antigen at 31.5 kDa, which gave ELISA values, 100% sensitivity and 99% specificity (12 diseases). Only one of fascioliasis showed a false positive [10]. Besides, enzymes in the excretory-secretory products of lung flukes can also be useful in the serodiagnosis of paragonimiasis westermani, as shown for cysteine protease antigens in an IgG-ELISA [16]. In another study was a chicken cystatin capture ELISA for detection of antibodies to *P. westermani*. In this assay, the cystatin binds with fluke cysteine proteinases in excretory-secretory products, and then is exposed to sera from paragonimiasis patients containing antibodies to the cycteine proteases. The paragonimiasis cystatin capture ELISA showed high reactivity [17], although native cysteine proteinases are highly conserved and possess common epitopes that are shared among many *Paragonimus* species including *P. westermani*, *P. miyazakii* and *P. ohirai* [16], thereby limiting their immunodiagnostic potential.

In addition to recombinant technology allowing the production of an unlimited amount of antigen, its application in generating a DNA recombinant protein of *P. westermani* eggs resulted in a significant increase in the sensitivity and specificity of an ELISA for paragonimiasis diagnosis to 90.2 and 100%, respectively, and also useful for an epidemiological study of paragonimiasis [18]. A recently developed IgG-ELISA using the recombinant cysteine protease antigen (21 kDa) derived from cDNA of the *Paragonimus skrjabini* juvenile stage was reported to have a sensitivity of 95.5% and no cross-reaction with antibodies from the other human diseases in the study by Yu et al. [19], i.e. echinococcosis granulosus, taeniasis solium, schistosomiasis japonicum and trichinellosis spiralis [19]. Another recombinant antigen is the 35 kDa cathepsin L lacking signal peptide (rPpsCatL), which is produced from the cDNA of *P. pseudoheterotremus* worms and belongs to the cysteine protease group. This rPpsCatL was used in IgG-ELISA for paragonimiasis heterotremus, resulting in 100% sensitivity and 95.6% specificity (27 diseases). Cross-reactivity occurred from ten cases of strongyloidiasis, toxocariasis, Brugian filariasis, ascariasis, opisthorchiasis and fascioliasis [13]. Despite the fact that the recombinant antigens used in that study (rPpsCatL) and the current study (proactivator polypeptide) possessed the same molecular weight, the sensitivity and specificity results for their corresponding IgG-ELISAs suggest that they differ in antigenic reactivity to antibodies. However, both of these recombinant antigens were cross-reactive with antibodies from strongyloidiasis and opisthorchiasis. Further studies of the *P. heterotremus* proactivator recombinant antigen could lead to improvements in its diagnostic specificity and sensitivity. For example, identification and immune testing of individual antibody-reactive epitopes through epitope mapping of the protein could lead to highly-specific single-epitope diagnostic testing formats. Currently we are exploring this and other, serodiagnostic tests for the detection of ectopic human paragonimiasis infections.

## Conclusions

In the present study, a novel recombinant protein antigen was produced from the cDNA of *P. heterotremus* worms. This antigen specifically responded to antibody directed against the specific diagnostic band of native 35 kDa antigen. The insert of cDNA is suggested to be homologous to a proactivator polypeptide. The IgG-ELISA was used to evaluate this recombinant protein and this resulted in sensitivity, specificity, and positive and negative predictive values of 88.89, 95.51, 74.42 and 98.32%, respectively. False positives were found from only a few cases of four helminthic infections and one protozoan infection. In contrast to diagnoses, symptoms and history of intermediate host consumption are helpful for physicians to predict early stages of disease. However, for clinically unpredicted or suspected cases of paragonimiasis, which may result from immature or mature *Paragonimus* worms located outside the lungs, serodiagnosis can be of great assistance as an adjunct diagnostic tool. Therefore, the incorporation of the recombinant proactivator polypeptide as a serological test antigen shows promise for the detection of human paragonimiasis.

## Additional files

Additional file 1: **Figure S1.** Sizes of the inserted cDNA in randomly selected clones from the *P. heterotremus* cDNA library. PCR products resulting from amplification with the pJET forward primer and pJET reverse primer were separated on a 1.2% agarose gel. The sizes of inserted cDNA fragments from random clones (Lanes 1–14) were determined by comparison with the Kapa universal ladder (Lane M). (TIF 1102 kb)

Additional file 2: **Figure S2.** The sizes of CE3 PCR products. PCR was performed on CE3 and the resulting products were run on a 1.2% agarose gel. Lane M: marker; Lane 1: CE3 clone. (TIF 780 kb)

Additional file 3: **Figure S3.** Overview of clone CE3. DNAMan software was used to produce a schematic overview of clone CE3. (TIF 405 kb)

## Abbreviations

ABTS: 2,2-azino-di-(3-ethyl-benzthiazoline sulfonate)
AUC: area under the ROC curve
CI: confidence interval
ELISA: enzyme-linked immunosorbent assay
His6: histidine 6
IgG: immunoglobulin G
IP: intraperitoneal route
IPTG: isopropyl-l-thio-β-D-galactopyranoside
Ni-NTA: nickel-nitrilotriacetic acid
OD: optical density
PBS: phosphate-buffered saline
ROC: receiver operating characteristic
rPpsCatL: recombinant *Paragonimus pseudoheterotremus* cathepsin L
SDS-PAGE: sodium dodecyl sulphate-polyacrylamide gel electrophoresis
