# Supplementary figures and images for: ELISA based on a recombinant Paragonimus heterotremus protein for serodiagnosis of human paragonimiasis in Thailand
Source: Parasit Vectors. 2018 May 30;11:322. doi: 10.1186/s13071-018-2878-5 (PMC5975669; doi:10.1186/s13071-018-2878-5)

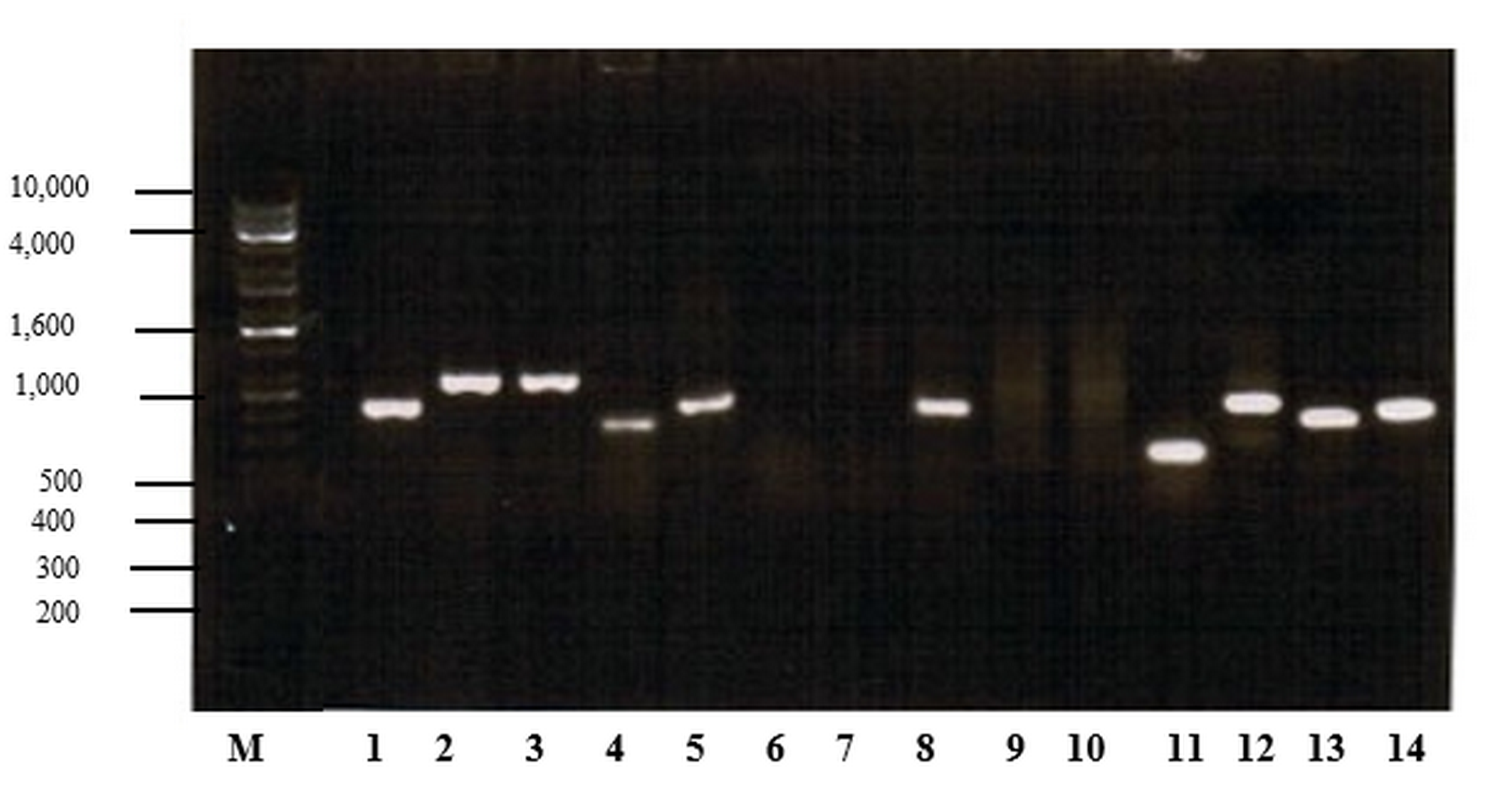

Supplement: Supplementary file 1 — Figure S1. Sizes of the inserted cDNA in randomly selected clones from the P. heterotremus cDNA library. PCR products resulting from amplification with the pJET forward primer and pJET reverse primer were separated on a 1.2% agarose gel. The sizes of inserted cDNA fragments from random clones (Lanes 1–14) were determined by comparison with the Kapa universal ladder (Lane M). (TIF 1102 kb) [file 13071_2018_2878_MOESM1_ESM.tif]

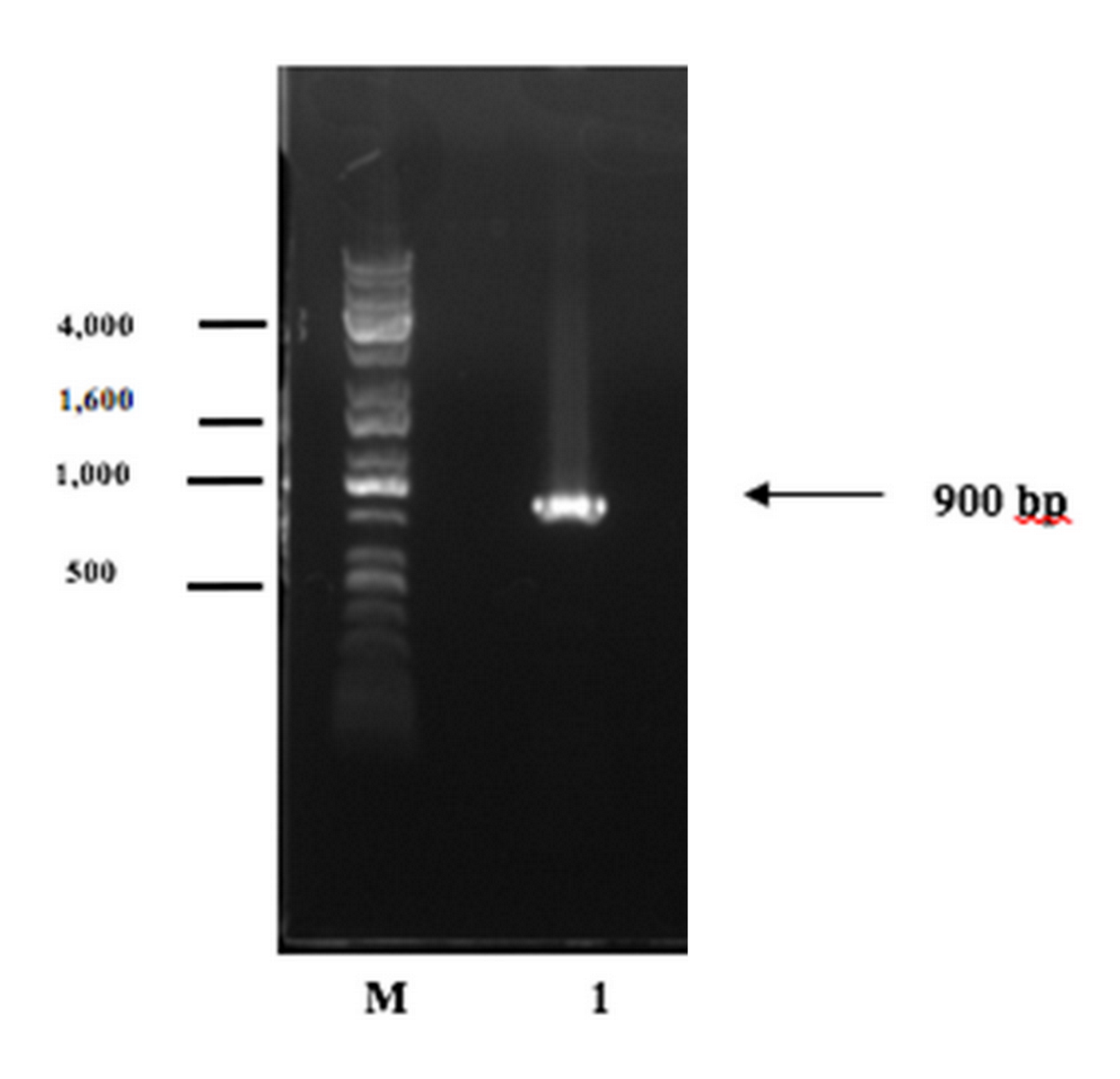

Supplement: Supplementary file 2 — Figure S2. The sizes of CE3 PCR products. PCR was performed on CE3 and the resulting products were run on a 1.2% agarose gel. Lane M: marker; Lane 1: CE3 clone. (TIF 780 kb) [file 13071_2018_2878_MOESM2_ESM.tif]

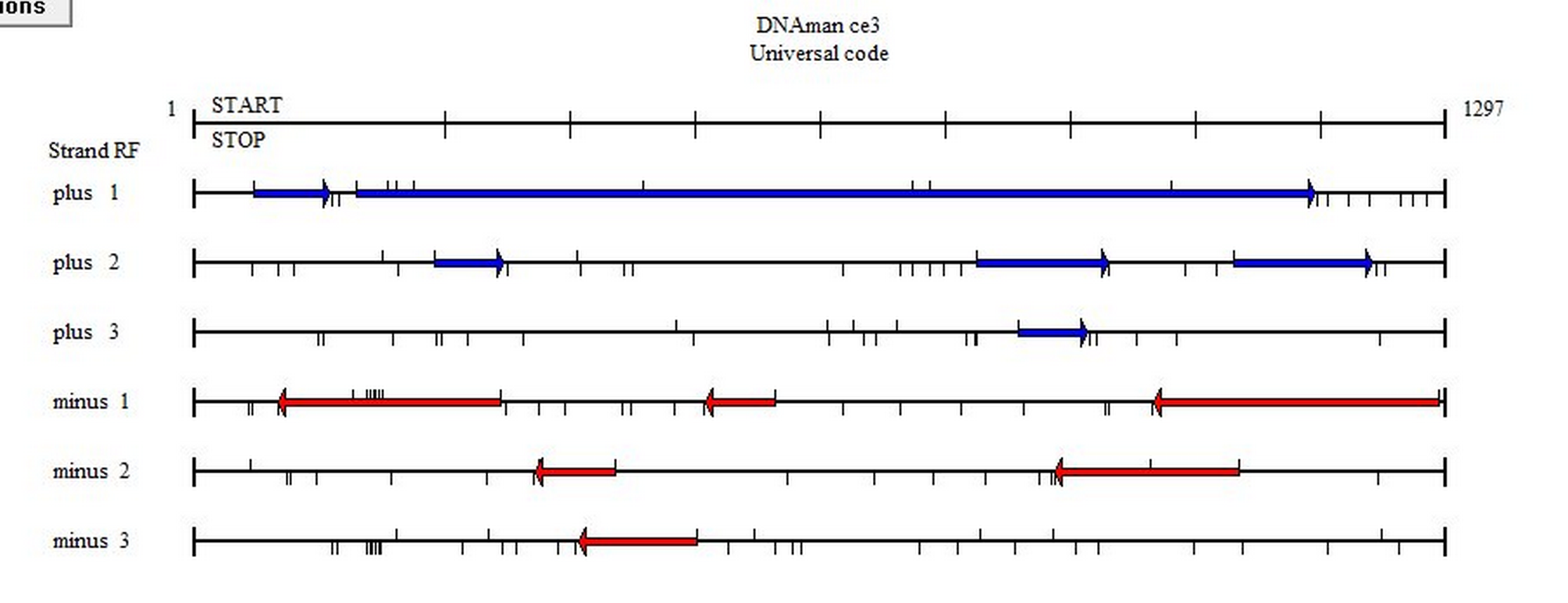

Supplement: Supplementary file 3 — Figure S3. Overview of clone CE3. DNAMan software was used to produce a schematic overview of clone CE3. (TIF 405 kb) [file 13071_2018_2878_MOESM3_ESM.tif]
